# *FaRCa1*: a major subgenome-specific locus conferring resistance to *Colletotrichum acutatum* in strawberry

**DOI:** 10.1007/s00122-018-3263-7

**Published:** 2018-12-18

**Authors:** Natalia Salinas, Sujeet Verma, Natalia Peres, Vance M. Whitaker

**Affiliations:** 1 Department of Horticultural Sciences, University of Florida, IFAS Gulf Coast Research and Education Center, Wimauma, FL 33598 USA; 2 Department of Plant Pathology, University of Florida, IFAS Gulf Coast Research and Education Center, Wimauma, FL 33598 USA

## Abstract

Optimal strategies for genetic improvement in crops depend on accurate assessments of the genetic architecture of traits. The overall objective of the present study was to determine the genetic architecture of anthracnose fruit rot (AFR) resistance caused by the fungus *Colletotrichum acutatum* in the University of Florida strawberry (*Fragaria *× *ananassa*) breeding germplasm. In 2016–2017, 33 full-sib families resulting from crosses between parents with varying levels of AFR resistance were tested. In 2017–2018, six full-sib families resulting from putative heterozygous resistant parents and homozygous susceptible parents were tested. Additionally, a validation population consisting of 77 advanced selections and ten cultivars was tested in the second season. Inoculation was performed using a mixture of three local isolates of the *C. acutatum* species complex. Phenotypes were scored weekly, and genotyping was performed using the IStraw35 Affymetrix Axiom^®^ SNP array. A pedigree-based QTL analysis was performed using FlexQTL™ software. A major resistance locus, which we name *FaRCa1*, was detected in both seasons with a peak located at 55–56 cM on LG 6B and explaining at least 50% of the phenotypic variation across trials and seasons. The resistant allele exhibited partial dominance in all trials. The *FaRCa1* locus is distinct from the previously discovered *Rca2* locus, which mapped to LG 7B. While *Rca2* is effective against European isolates from pathogenicity group 2, *FaRCa1* appears to confer resistance to isolates of pathogenicity group 1.

**Electronic supplementary material:**

The online version of this article (10.1007/s00122-018-3263-7) contains supplementary material, which is available to authorized users.

## Introduction

Optimal strategies for genetic improvement in allo-octoploid (2*n* = 8*x* = 56) strawberry (*Fragaria *× *ananassa*) depend on the genetic architecture of target traits (Whitaker et al. 2012). Accurate estimations of heritability and of the number, locations and effects of loci controlling traits are the basis for strategic decisions in breeding programs (Moose and Mumm 2008). For efforts to discover and characterize genetic loci, three characteristics are important: population type, statistical methodology and molecular resources available for a given species.

Biparental segregating populations have been predominantly utilized for QTL analysis in cultivated strawberry. Economically important fruit quality traits (Lerceteau-Kohler et al. 2012; Zorrilla-Fontanesi et al. 2012) and flowering traits (Weebadde et al. 2007) in strawberry have been described using biparental populations. Loci associated with disease resistance in strawberry have also been described using biparental populations, including for Verticilium wilt caused by *Verticillium dahliae* (Antanaviciute et al. 2015), anthracnose caused by *Colletotrichum acutatum* (Lerceteau-Kohler et al. 2005), crown rot caused by *Phytophthora cactorum* (Denoyes-Rothan et al. 2004) and red stele root rot caused by *Phytophthora fragariae* (Haymes et al. 2000). However, limitations inherent to biparental populations can restrict the applicability of these results in breeding programs (van de Weg et al. 2004; Bink et al. 2014). Trait-associated loci and their effects, while significant within a single cross, might not be relevant for broader germplasm. Most fruit breeding programs are characterized by many pedigree-connected clones, complex multi-generational families with variable numbers of progeny and diverse genetic backgrounds (Bink et al. 2002, 2012). To detect loci and their effects across representative genetic backgrounds, it is advantageous to simultaneously analyze pedigree-connected families that represent the full genetic variability of a population (Verma et al. 2017b). Utilizing complex, diverse populations in QTL detection provides additional advantages such as the exploitation of historical recombination events, higher mapping resolution and greater sensitivity for detection of loci with small phenotypic effects (Xu et al. 2017).

Recent improvements in statistical methods have been critically important for mapping studies, especially for analysis of complex population structures. Bayesian analysis methods with Markov chain Monte Carlo algorithms are flexible and powerful tools that use bi-allelic or multi-allelic QTL models to estimate chromosomal location, number, mode and magnitude of QTLs in unbalanced population sets (Bink et al. 2002, 2008, 2014). Moreover, the Bayesian approach is appropriate when marker information is incomplete due to use of dominant markers (Bink et al. 2002). The software package FlexQTL™ utilizes a Bayesian pedigree-based analyses (PBA) approach for QTL discovery and characterization via the software Pedimap (Voorrips et al. 2012) and Visual FlexQTL™ (van de Weg et al. 2004, 2015) which support the graphical representation of FlexQTL™ outputs (van de Weg et al. 2004). Loci for economically important traits have been characterized using this software in fruit crops including sweet cherry (Rosyara et al. 2013; Cai et al. 2017), peach (Fresnedo-Ramirez et al. 2015, 2016; Hernández-Mora et al. 2017a, b) and apple (Allard et al. 2016; Di Guardo et al. 2017; Durand et al. 2017; Howard et al. 2017; van de Weg et al. 2017).

FlexQTL™ has also been recently utilized in octoploid strawberry in concert with subgenome-specific genetic maps. Four biparental crosses were assessed to detect *FaRXf1* (LG 6D), a major locus that confers resistance to angular leaf spot (ALS) caused by *Xanthomonas fragariae* (Roach et al. 2016). A large-effect QTL, *FaRPc2* (LG 7D) for resistance to crown rot, caused by *Phytophthora cactorum*, was characterized in many genetic backgrounds using 139 full-sib families obtained from 61 parents in the UF strawberry breeding germplasm (Mangandi et al. 2017). A moderate-effect QTL, *FaRCg1* (LG 6B), explained most of the genetic variance for resistance to crown rot caused by *Colletotrichum gloeosporioides* in 62 full-sib families in the University of Florida (UF) strawberry breeding program (Anciro et al. 2018). Furthermore, QTLs were detected for soluble solids content (SSC) (LG 6A), fruit size (LG 2BII), pH (LG 4CII), titratable acidity (LGs 2A and 5B) and perpetual flowering (LG 4A) in 23 pedigree-connected families obtained from 11 parents that represent the breeding populations included in the RosBREED project (Iezzoni et al. 2017; Verma et al. 2017b). These results have enabled a robust platform for marker-assisted selection for *FaRXf1* and *FaRPc2* and future applications for other QTLs present in the UF strawberry breeding program and other strawberry breeding programs (Whitaker et al. 2017).

One of the major challenges in strawberry genetics has been the availability of high-throughput, subgenome-specific markers. Since the cultivated strawberry has four subgenomes, each locus can represent up to eight different alleles in a single individual, making it difficult to accurately analyze segregation. Initial progress began with the availability of a reference genome for the diploid (2*n* = 2*x* = 14) *Fragaria vesca* ssp. *vesca* (Shulaev et al. 2011), an ancestor of the cultivated strawberry *Fragaria *× *ananassa* (Tennessen et al. 2014). Based on the alignment of short-read sequences from 19 strawberry accession to the diploid strawberry reference genome, di-allelic and multi-allelic single-nucleotide polymorphisms (SNPs) and indels were incorporated into the 90K Affymetrix^®^ Axiom^®^ array, the first high-throughput genotyping platform for cultivated strawberry (Bassil et al. 2015). The applicability of the 90K Affymetrix^®^ Axiom^®^ array was limited in breeding programs due to high cost per sample (Bassil et al. 2017), and a subset of high-quality genetically mapped markers was chosen for inclusion on the 38K Axiom^®^ IStraw35 384HT array (Verma et al. 2017a). These arrays now provide an abundance of subgenome-specific SNPs for use in genetic studies.

Anthracnose diseases caused by *C. acutatum* are economically impactful in strawberry nurseries and fruiting fields worldwide, but particularly in Florida, where warm and wet conditions provide the pathogen with the ideal environment to cause economic losses (Howard et al. 1992). Growth of *C. acutatum* is most favorable between 24 and 28 °C (Smith 1990) and during continuous periods of wetness (Forcelini et al. 2017). Such conditions are common during the strawberry production season in Florida. The fungus can survive as appressoria and produce quiescent infections on strawberry leaves from nursery plants where symptoms are usually not observed (Peres et al. 2005). In some cases, infected runner plants might show symptoms on petioles and roots (Mertely and Peres 2012). In the field, *C. acutatum* spores are dispersed by irrigation, rain, insects and workers (Mertely et al. 2017). Overhead irrigation used to facilitate bare-root transplant establishment is also an important form of dispersal. Symptoms can be observed in runners, crowns, leaves, flowers and fruit. Small black or dark brown spots can be found on green fruit. In ripening fruit, lesions are firm, dark brown and sunken (Mertely et al. 2017). Lesions harbor developing conidia and make fruit unmarketable. Fruit disease caused by *C. acutatum* is commonly known as anthracnose fruit rot (AFR) in the USA and as black spot in the UK (Simpson et al. 1994; Mertely et al. 2017).

Chemical control of AFR is the most common practice in commerce. The protectant multi-site fungicide captan and single-site fungicides within the quinone outside inhibitors (QoI) such as azoxystrobin and pyraclostrobin in the commercial products Abound^®^, Cabrio^®^, and the phenylpyrrole (PP) fludioxonil in Switch^®^ are widely used in Florida for anthracnose management (Mertely et al. 2017). However, *C. acutatum* isolates resistant to QoI fungicides were reported in 2013, limiting chemical management of AFR primarily to captan sprays (Forcelini et al. 2016).

For many decades, recurrent phenotypic selection has been applied in the UF strawberry breeding program to improve fruit quality traits (Whitaker et al. 2012) and disease resistance, including AFR. Previous studies have documented broad variability for resistance to AFR in UF breeding germplasm, with most cultivars being moderately to highly resistant (Chandler et al. 2006; Seijo et al. 2008, 2012a, b). While phenotypic selection has been historically effective, it has been less so in recent decades. Summer nurseries have moved out of Florida to temperate locations where conditions are less favorable for Colletotrichum diseases, and fungicides are increasingly used in the UF breeding plots to reduce incidence of several diseases according to commercial practices. As a result, the frequency of resistant genotypes in the UF breeding population has decreased in recent years (unpublished data). Moreover, the ideal scenario for phenotypic selection for disease resistance requires considerable resources for inoculated trials (Moose and Mumm 2008). It would be highly advantageous to utilize molecular tools in breeding for AFR resistance. However, the genetic architecture of AFR resistance in the UF breeding program must first be described.

Previous European research demonstrated that a dominant allele at the single locus *Rca2* controls high-level resistance to *C. acutatum* isolate 688b from pathogenicity group (PG) 2 in strawberry (Denoyes-Rothan et al. 2005). Five other QTLs were found to give moderate resistance to the *C. acutatum* isolate 494a, which was assigned to PG-1, in a cross between ‘Capitola’ and CF1116 (Denoyes-Rothan et al. 2004). Since the *Rca2* resistance allele was reportedly present in UF germplasm (Denoyes-Rothan et al. 2005), it was originally assumed that *Rca2* was the main resistance source in the UF breeding population. However, molecular markers linked to *Rca2*, which give resistance to *C. acutatum* isolate 688b and 1267b from PG-2 (Lerceteau-Kohler et al. 2005), do not explain resistance/susceptibility to AFR in UF cultivars (unpublished data). UF cultivars are a source for AFR resistance in other breeding programs worldwide.

The overall objective of the present study was to determine the genetic architecture of AFR resistance in UF strawberry breeding germplasm using *C. acutatum* isolates collected in Florida. Specific objectives were to: (1) phenotypically characterize AFR resistance in UF breeding germplasm in inoculated field trials, (2) detect subgenome-specific chromosome regions associated with resistance to AFR using pedigree-based QTL analysis in complex, multi-parental populations representing the diversity of UF breeding germplasm, and (3) validate the presence and phenotypic effects of loci associated with resistance to AFR in the parent pool of the UF strawberry breeding program and in widely grown cultivars.

## Materials and methods

### QTL discovery and validation populations

A large, multi-parental population set was evaluated in each of two consecutive strawberry seasons. All crosses included were representative subsets of crosses made in the UF breeding program each year, each parent arising from an elite breeding population that has undergone phenotypic recurrent selection for more than 15 generations. The 2016–2017 population set consisted of 33 full-sib families from crosses among 31 parents (Supplementary Fig. 1). Parents were chosen to represent a wide range of disease phenotypes, with resistant × resistant, resistant × susceptible and susceptible × susceptible crosses included. The 2017–2018 discovery population set consisted of six full-sib families from crosses made among seven parents (Supplementary Fig. 2). These crosses were resistant × susceptible and predicted to segregate 1:1 based on 2016–2017 results. From each full-sib family, 4 to 26 seedlings in 2016–2017 (Supplementary Fig. 1) and 42 to 68 seedlings in 2017–2018 (Supplementary Fig. 2) were randomly selected for inclusion in the study.

Seeds from each cross were germinated at the UF Gulf Coast Research and Education Center (GCREC) in Wimauma, Florida, in April of each year, transplanted into individual peat pellets and transported to the breeding program’s summer nursery located in southern Oregon in June of each year for clonal replication. Runner plants (clonal replicates) from each seedling were allowed to root, then collected from the nursery as bare-root transplants in September and transported to Florida for evaluations at the GCREC research farm. Four clonal replicates of each individual were planted in a randomized complete block field design (RCBD) with one runner plant in each of four blocks, each block consisting of two raised beds. Eight clonal replicates of each parent and check genotype were planted, one in each bed. Transplanting took place on Oct 10, 2016, and Oct 10, 2017.

Eighty-seven individuals consisting of elite selections (77) representing the parent pool of the breeding program and cultivars (10) were evaluated for resistance to AFR during the 2017–2018 season in a separate inoculated trial (Supplementary Table 1). For each selection or cultivar, four plants were planted in each of two blocks (eight total clonal replicates) in a randomized complete block (RCB) design with each block consisting of one planting bed. Transplanting took place on Oct 10, 2017.

### Inoculation and disease rating

Isolates 02-163, 02-179 and 03-32 (recently described as *C. nymphaeae* within the *C. acutatum* species complex, N. Peres unpublished data) were, respectively, obtained from petiole, fruit and crowns of symptomatic strawberry plants submitted to the GCREC plant disease diagnostic clinic from strawberry farms located in central Florida (MacKenzie et al. 2009). Isolates 02-163, 02-179 and 03-32 were obtained from cultivars Festival, Aromas and Treasure, respectively, in fall 2002 and 2003. These three isolates showed high anthracnose incidence and severity on detached strawberry fruit from cultivar Camarosa (MacKenzie et al. 2009). Inoculum was produced by growing the three isolates separately on potato dextrose agar (PDA) at room temperature with constant light for 7 days until sporulation. Plates were flooded with sterile-distilled water when growth turned orange and reached near the border of the plate. A spore suspension for each isolate was passed through cheesecloth to remove dislodged mycelia. A final suspension was adjusted to 1 × 10^5^ conidia/ml by combining equal concentrations of each isolate. Each plant was inoculated with approximately 5 ml of the final conidia suspension. Inoculations were performed late in the afternoon using a handheld sprayer by placing the nozzle of the sprayer approximately 2 cm above the crown. For the 2016–2017 trial, two inoculations were performed, the first one on Oct 21, 2016, and the second one on Dec 5, 2016. For the 2017–2018 trial, inoculation was performed on Nov 7, 2017. Overhead irrigation was applied after inoculation to ensure leaf wetness for at least 4–6 h. AFR incidence was recorded weekly for 11 weeks starting 12 weeks after the first inoculation in the 2016–2017 trial, and for 8 weeks starting 15 weeks after inoculation in the 2017–2018 trial. All ripe fruits were harvested weekly from all plants, and each fruit was examined visually for AFR symptoms. A fruit was considered diseased regardless of the proportion of the fruit surface affected. The proportion of symptomatic to total fruit harvested was determined for each plant (clonal replication). A mean incidence was calculated across replications for each individual.

### DNA extraction and SNP genotyping

For the discovery populations and validation population in both years, 30–60 mg of unexpanded leaf tissue from each individual was collected into 96-well plates and frozen at − 80 °C until extraction. Immediate parents and other pedigree-connected individuals with discovery populations were also included. DNA extraction was performed using a modified Integrated DNA Technologies (IDT) Plant DNA Extraction Protocol (Keb-Llanes et al. 2002). Prior to DNA extraction, frozen samples were ground with a Fisher Scientific PowerGen high-throughput homogenizer (Pittsburgh, PA) twice for 1 min with a 3-min refreezing at −  80 °C between grindings. Genotyping  was performed using the 38K Axiom^®^ IStraw35 384HT array (Verma et al. 2017a). Genotyping errors were detected by comparing, within full-sib families, SNP calls of each individual with that of the parental genotypes, and replaced with ‘no call’ if not matching possible genotypes. If the correct parent was not obvious, the incorrect parent was replaced with an ‘undetermined’ parent. In addition, markers with inheritance errors, as determined in the ‘mconsistency’ file of FlexQTL™ outputs, were removed from the data and the data re-analyzed in FlexQTL™ until errors were minimized.

### Linkage mapping

Genetic maps were constructed in JOINMAP 4.1 software (Van Ooijen 2006, 2011) using 165 progeny from a cross between ‘FL_08-10’ × ‘12.115-10’ and 91 progeny from ‘Winter Dawn’ × ‘Treasure.’ The markers were curated to achieve consistent performance across the germplasm, to maximize polymorphism and to exclude markers with fraction values of observed (oDR) and expected (eDR) double recombination singletons > 0.05 (Clark et al. 2014). Subgenomes and orientations of linkage groups were assigned according to van Dijk et al. (2014) as the maps share many common markers with those described in Bassil et al. (2015) and Mangandi et al. (2017). The ‘FL_08-10’ × ‘12.115-10’ map utilized for QTL analyses was the same map used in the discovery of *FaRCg1* which confers resistance to *C. gloeosporioides*, the causal agent of Colletotrichum crown rot in strawberry (Anciro et al. 2018). After initial QTL analysis runs, the map was further curated using graphical genotyping to remove singletons and unrealistic double recombination events to resolve genetic distances for linkage groups with significant QTL. The mapping and curation process attempted to maximize polymorphism in chosen markers. The ‘Winter Dawn’ × ‘Treasure’ map was used to locate the previously published *Rca2* locus, since the original report used an AFLP map that could not be related to current SNP-based maps. This map was utilized since the *Rca2*-associated CAC_240 marker (Lerceteau-Kohler et al. 2005) segregated 1:1 in this population and did not segregate in ‘FL_08-10’ × ‘12.115-10.’

### GWAS and QTL analyses

Preliminary genome-wide association studies (GWAS) for all populations were performed using the GAPIT R package (Lipka et al. 2012) and ~ 38K polymorphic SNP markers anchored to the *F. vesca* spp*. bracteata* genome assembly (Tennessen et al. 2014) to survey the genome for regions that may be associated with AFR resistance. Pedigree-based QTL analyses with markers mapped to all subgenomes were performed using a Markov chain Monte Carlo (MCMC)-based Bayesian analysis in FlexQTL™ using a model with additive QTL effects and a maximum number of QTL of 20. The prior number of QTL was set to 1 (Bink et al. 2014), and genome-wide analyses were performed. Each analysis was performed with different starting seeds to create independence between iterations, using simulation chain lengths of 100,000 or 200,000 iterations with thinning values of 100 or 200, respectively. The effective sample size in the parameter file was set to 100. Each of the two iterations converged (effective chain samples, or ECS ≥ 100 for each of the parameters mean, variance of the error, number of QTL and the variance for the number of QTL) as recommended by Bink et al. (2014). In addition to seedlings and immediate parents, pedigree-connected cultivars and selections were also included in the analysis.

Two times the natural log of Bayes factors (BF) generated from genome-wide FlexQTL™ analysis was used to determine the total number of QTL (2lnBF_10_ ≥ 5) as well as QTL positions on individual LGs (Rosyara et al. 2013; Bink et al. 2014; Guan et al. 2015). Narrow-sense heritability (*h*^2^) was calculated by using statistical inferences from FlexQTL™ software outputs with the formula:

$$h^{2} = \frac{{{\text{VP}} - {\text{VE}}}}{\text{VP}}$$

where VP is the phenotypic variance of the trait and VE is the residual error variance (Bink et al. 2014). The proportion of phenotypic variation explained (PVE) by a QTL was calculated using the formula:

$${\text{PVE }} = {\text{ }}\left( {\frac{{{\text{wAVt}}}}{{{\text{VP}}}}} \right) \times 100$$

where wAVt is the weighted additive variance of the trait, adjusted for the portion of the variance explained by the QTL on a particular chromosomal position (obtained after post-QTL analysis), and VP is the total phenotypic variance of the trait.

### QTL validation via single-marker analyses

SNPs spanning the QTL region detected in the discovery populations were used in single-marker analyses to independently determine their association with AFR in the validation population. Single-marker analyses were performed for 19 markers using one-way ANOVA in R software (R Core Team 2013). The 19 Axiom probe sequences for these SNP markers were located to the diploid woodland strawberry *Fragaria vesca* genome assembly v4.0.a1 (Edger et al. 2018) via local BLAST in order to determine the physical positions of the probes.

## Results

### Phenotypic segregation

**Fig. 1:**
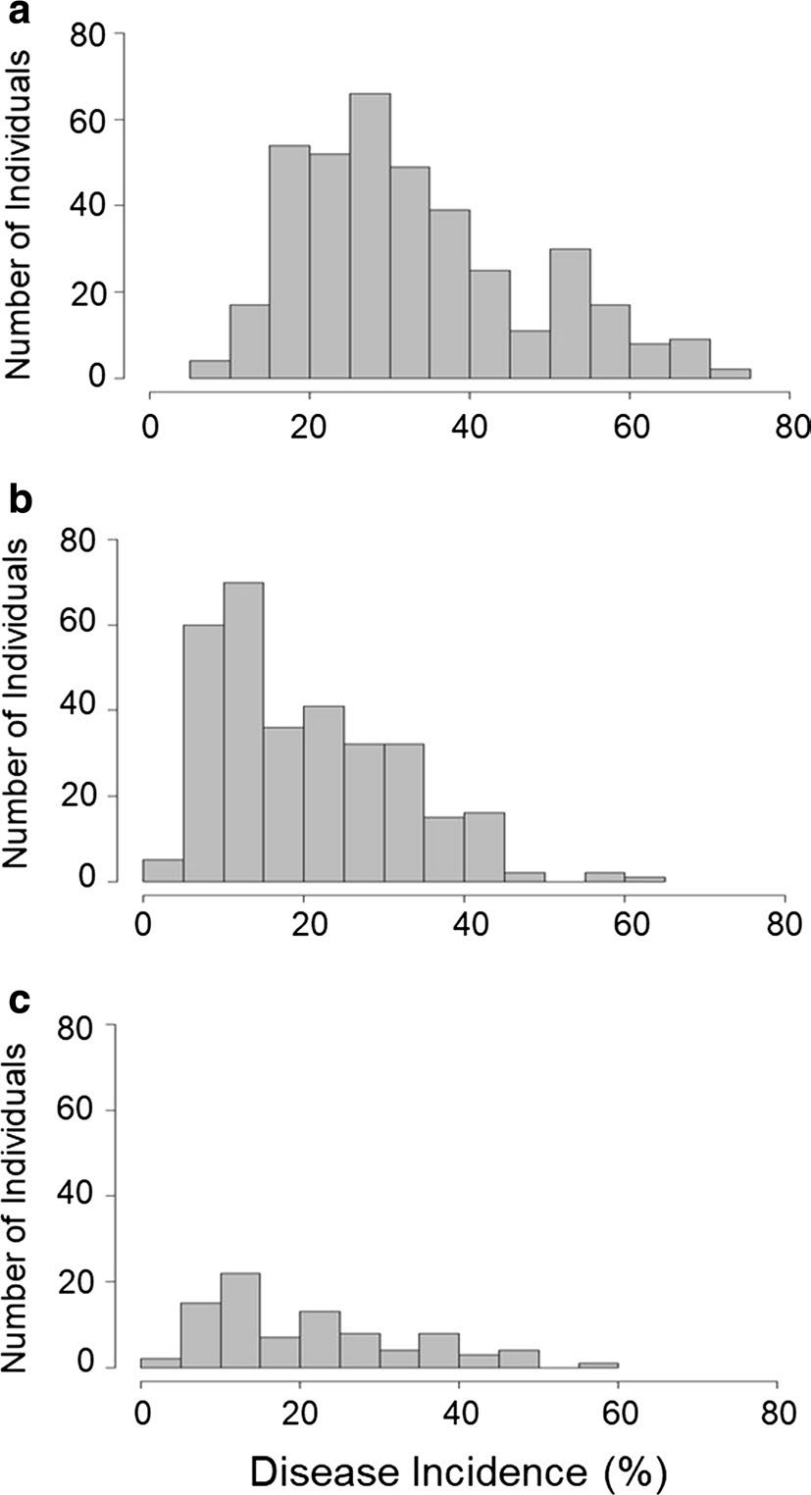
Frequency distributions of disease incidence for AFR caused by *C. acutatum* for **a** discovery population, 2016–2017; **b** discovery population, 2017–2018; and **c** validation population, 2017–2018

A wide range of phenotypic variability for resistance to AFR was observed in both seasons. Disease incidence for the discovery populations ranged from 6.4 to 72.3% and from 1.6 to 60.1% in the 2016–2017 and 2017–2018 seasons, respectively. Disease incidence for the validation population ranged from 3.4 to 59.4% in 2017–2018 (Fig. 1). Frequency distributions were strongly skewed toward higher values (susceptibility) in both discovery populations and validation population. Full-sib family means ranged from 21.1 to 47.2% in 2016–2017 and from 16.9 to 25.2% in 2017–2018. Means and standard errors for disease incidences of parents, selections and cultivars common in the two discovery populations in 2016–2017 and 2017–2018 and in the validation population in 2017–2018 are compared in Supplementary Table 2.

### Genotyping and linkage mapping

In the 2016–2017 and 2017–2018 discovery population sets, 28,044 and 23,438 polymorphic SNPs, respectively, were obtained. The subgenome-specific mapping and curation process resulted in a final map for ‘FL_08-10’ × ‘12.115-10’ of 9301 SNPs spanning 19.3 Morgan that maximized polymorphism across both discovery population sets (Anciro et al. 2018). In 2016–2017, the mean minor allele frequency (MAF) was 0.26, with 90% of markers having MAF between 0.05 and 0.5. In 2017–2018, the mean MAF was 0.22, with 75% of markers having MAF between 0.05 and 0.5. In 2016–2017, 89% of the 9301 SNPs were categorized as poly-high resolution, presenting all three possible SNP genotype classes (AA, AB and BB), 10% lacked one of the homozygous genotype classes (so-called no-minor homozygote markers), whereas the remaining 0.7% were homozygous. In 2017–2018, 61% of SNPs were poly-high resolution, 27% were no-minor homozygote markers, and the remaining 12% were homozygous. All retained markers were polymorphic in at least one of the population sets tested in a given season. Averages of MAF for individual LGs ranged from 0.09 (4A) to 0.41 (2C), and 0.07 (4A) to 0.38 (2D) in 2016–2017 and 2017–2018, respectively.

### QTL discovery

**Fig. 2:**
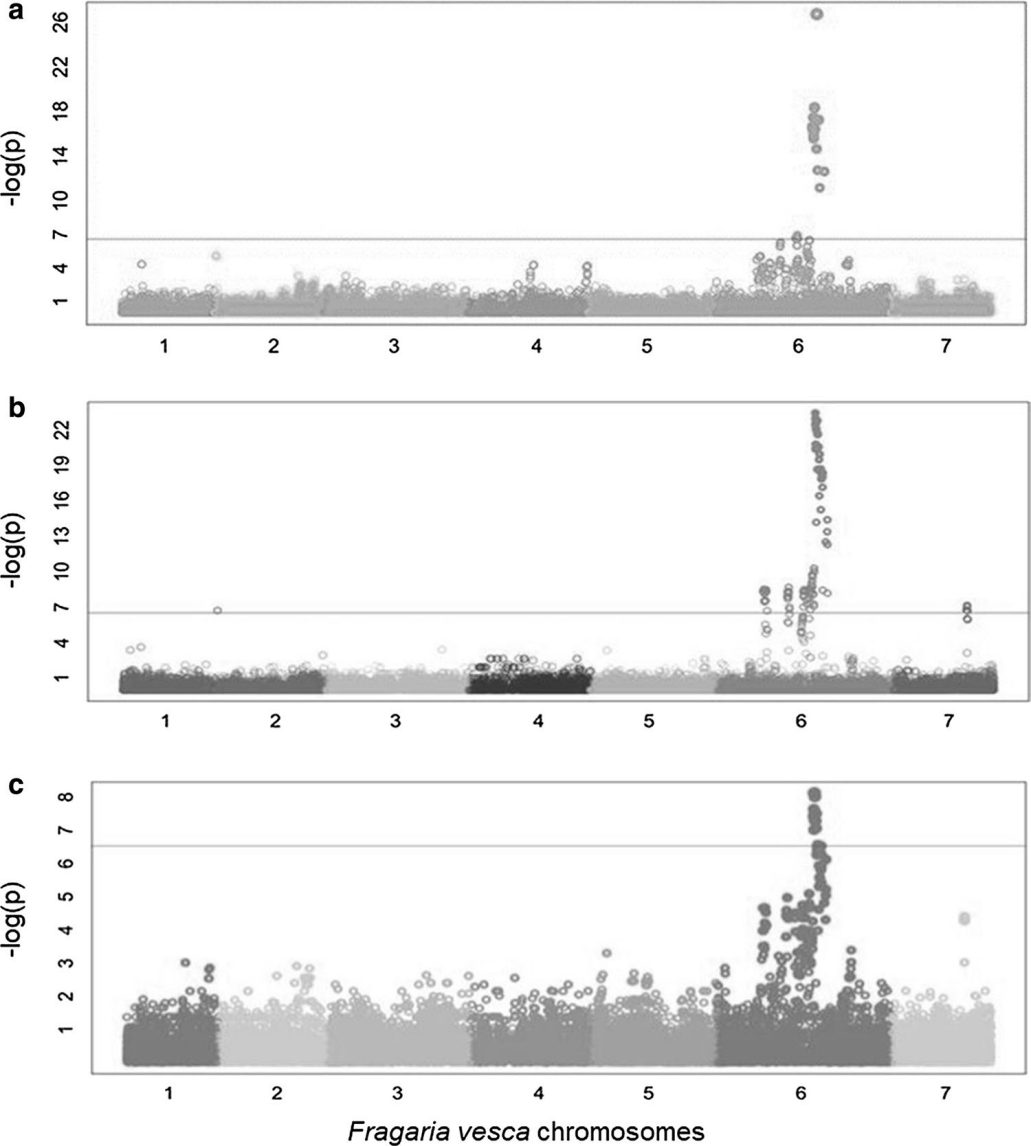
Graphical outputs from GWAS using 38,506 SNPs for discovery populations in 2016–2017 (**a**) and 2017–2018 (**b**) and a validation population in 2017–2018 (**c**) with SNP physical positions anchored to the *Fragaria vesca* spp. *bracteata* genome assembly

**Fig. 3:**
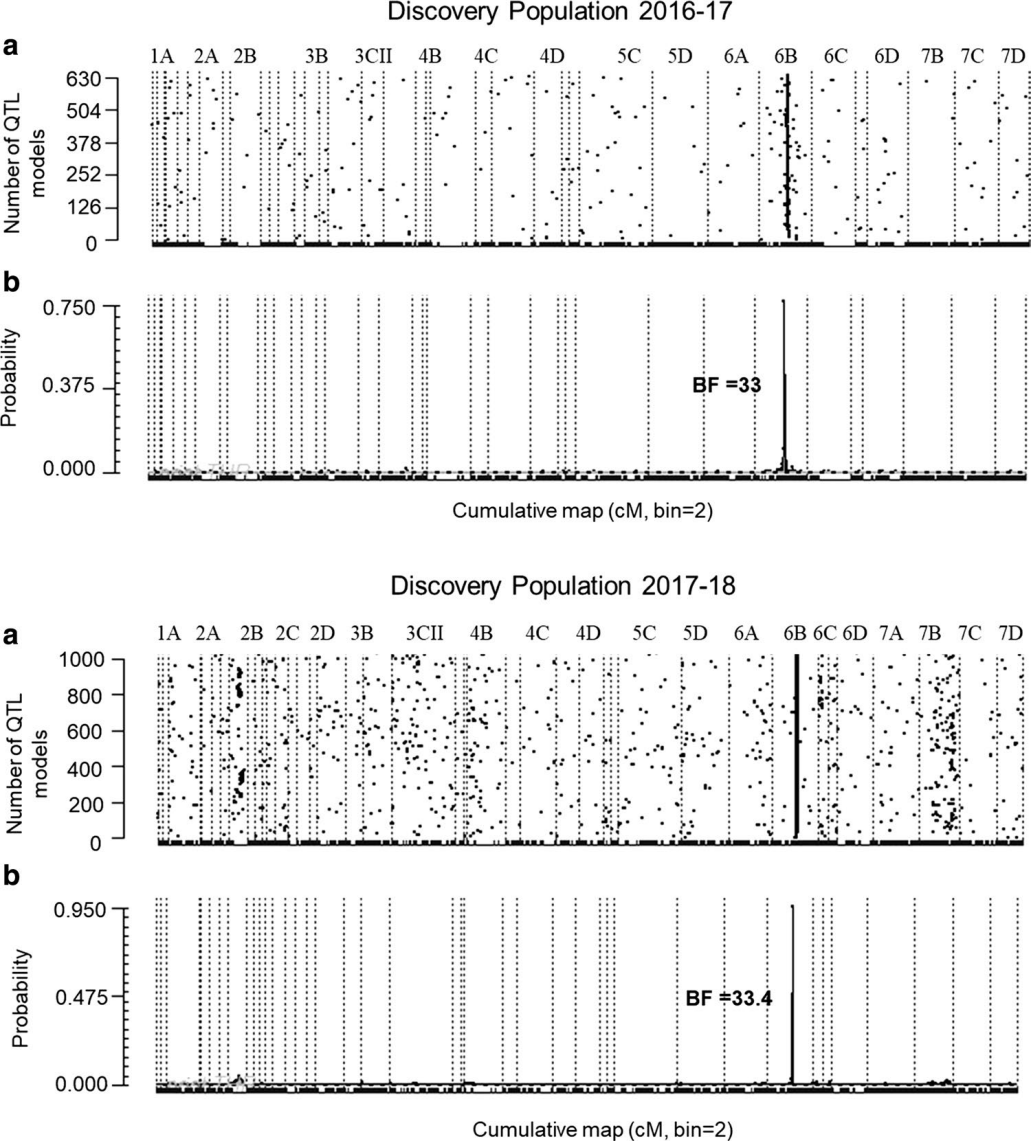
**a** Traces of the QTL models across 28 LGs. Each dot represents an iteration, and continuous dots that form a line confirm the presence of a QTL. **b** Posterior probability of the QTL position. When the Bayes factor (BF) is greater than 25, the evidence of presence of a QTL is considered decisive

Preliminary GWAS analyses for all populations showed highly significant evidence for a single locus on LG 6 associated with AFR incidence (Fig. 2). Subgenome-specific FlexQTL™ analyses for the 2016–2017 discovery population showed evidence for a one QTL model (2lnBF_10_ = 32.9) in independent MCMC simulations using 1 as prior number of QTL (Fig. 3). Four different runs using from 5 to 8 LGs also showed strong evidence for a one QTL model (2lnBF_10_ = 28–30.3) in the same location. The QTL interval was consistently located at 54–57 cM on LG 6B. The phenotypic variance explained (PVE) by this locus across five runs averaged 50.7% in 2016–2017, and narrow-sense heritability estimates for the five runs averaged 0.46. Genome-wide FlexQTL™ analysis results for the 2017–2018 discovery population were consistent with those obtained with the 2016–2017 discovery populations, with decisive evidence (2lnBF_10_ = 33.4) for one QTL on LG 6B (Fig. 3). The PVE in the second year was 94.3% with a narrow-sense heritability of 0.61.

The locus found in this study appears to be distinct from the previously discovered *Rca2* locus. Sequence-characterized amplified region (SCAR) markers linked to *Rca2* (Lerceteau-Kohler et al. 2005) were blasted to the *Fragaria vesca* diploid reference genome (Edger et al. 2018). Reverse primers for marker CAC_240_2 and CAC_417_3 blasted to a distal portion of chromosome 7, whereas forward primers for marker CAC_240_2 did not blast to any part of chromosome 7. Additionally, marker CAC_240_2 mapped to the end of LG 7B in ‘Winter Dawn’ × ‘Treasure.’ Thus, two lines of evidence place *Rca2* on a different chromosome from the novel locus described in this study.

### QTL validation

**Fig. 4:**
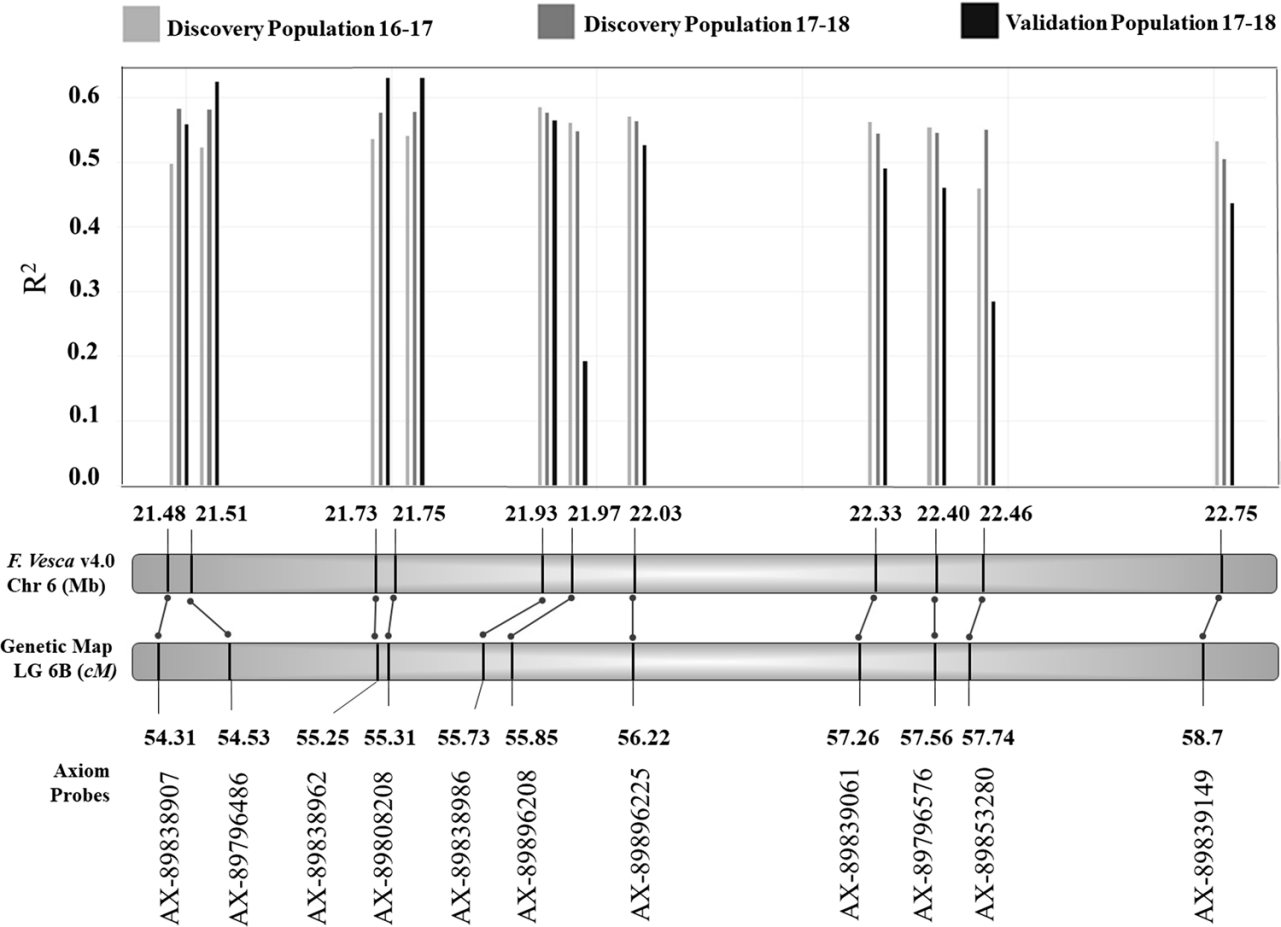
Single-marker analysis in two discovery populations and a validation population for 11 SNP markers located in the *FaRCa1* region. Six of the 19 SNP markers analyzed were highly monomorphic and thus not informative and two could not be confidently ordered on the linkage group

**Fig. 5:**
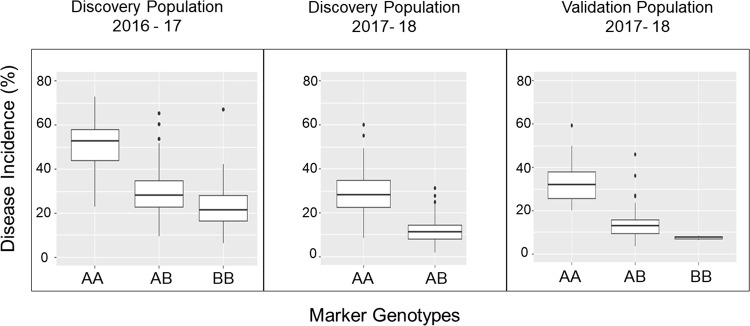
Box plots showing disease incidence by marker genotype for IStraw35 SNP probe AX-89838986

Associations for markers present in the QTL region were obtained for the discovery populations in seasons 2016–2017 and 2017–2018, and for the validation population in 2017–2018 (Fig. 4). Across all populations, the highest R-squared values correspond to SNP probes AX-89796486, AX-89838962, AX-89808208, AX-89838986 and AX-89896208 located between genetic position 54.53 cM and 55.85 cM on LG 6B of the genetic map. These positions correspond to the region between 21.51 and 21.97 Mb on chromosome 6 in the *F. vesca* reference genome. The SNP probe AX-89838986 had the highest average *R*-squared value across the two discovery populations and therefore was chosen as the most informative for examining allele interactions in all three populations (Fig. 5). In all cases, the heterozygous genotype aligns close to but not equal to the resistant homozygous genotype, indicating that resistance is conferred by a partially dominant allele at this locus.

In summary, our results demonstrate consistent evidence that a partially dominant allele at a large-effect locus near 55–56 cM on LG 6B confers resistance to three *C. acutatum* isolates from Florida. The effects of the locus were validated in a set of advanced selections and cultivars. Furthermore, the locus occurs at a different genetic location from the previously discovered anthracnose resistance locus *Rca2*. We therefore name this locus *FaRCa1* (*Fragaria Resistance* to *C. acutatum* locus 1).

## Discussion

The *FaRCa1* locus accounted for the genetic variation for AFR resistance observed in three field trials established across 2 years and inoculated with Florida isolates of *C. acutatum*. Together, the three trials represented the diversity of the UF elite breeding population. Leveraging historical recombinations and segregation among controlled crosses in large populations allowed the delineation of *FaRCa1* to a region of 460 kb with high confidence. Single-marker analyses indicated that SNP probes AX-89796486, AX-89838962, AX-89808208, AX-89838986 and AX-89896208 located in the interval 54.53–55.85 cM on LG 6B have the highest associations with AFR resistance. These positions correspond to the region between 21.51 and 21.97 Mb on chromosome 6 of the diploid woodland strawberry *F. vesca*. These results will form the initial basis for marker development and marker-assisted selection for *FaRCa1*.

Total phenotypic variation (PVE) and heritability estimates obtained from FlexQTL™ are genetic parameters inherent to the populations from which they were derived. Thus, differences in PVE (50.7% and 94.3%) and narrow-sense heritability (0.46 and 0.61) in the discovery populations of the first and second season are related to population structure and segregation of AFR. The discovery population in season 2016–2017 was composed of many crosses, some of which were resistant × resistant and susceptible × susceptible. In 2017–2018, putative heterozygous (Rr) resistant parents and homozygous (rr) susceptible parents were chosen to result in (1:1) segregation in each cross. Thus, the PVE should be larger in the second season, assuming a large effect for the locus. This was the case, despite the fact that the single inoculation and lower temperatures in 2017–2018 apparently limited disease progression in the field compared to 2016–2017, resulting in a lower overall AFR incidence in 2017–2018. Comparisons of disease incidence for parents, selections and cultivars in both seasons (Supplementary Table 2) reinforce the absence of genotype by environment interactions despite fewer symptoms observed in 2017–2018.

**Fig. 6:**
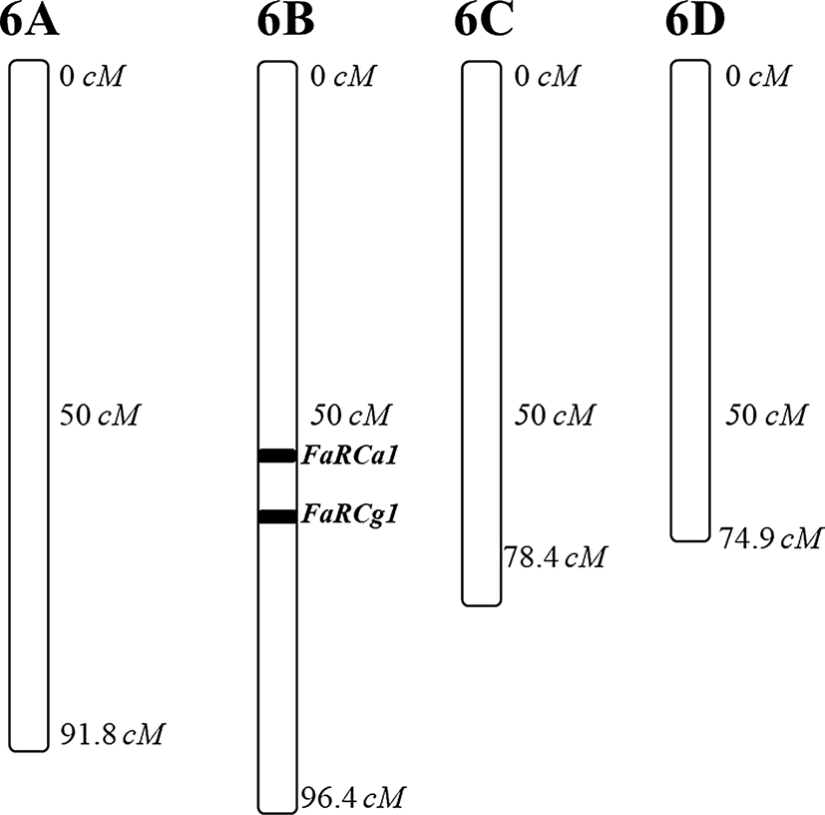
Schematic of LG 6 subgenomes based on a genetic map, showing the location of *FaRCa1* approximately 10 cM from *FaRCg1* on LG 6B

A separate locus, *FaRCg1,* was recently discovered that confers resistance to *C. gloeosporioides*, the causal agent of Colletotrichum crown rot in strawberry (Anciro et al. 2018). This locus mapped to 66–70 cM on LG 6B. Therefore, *FaRCg1* is located approximately 10 cM from *FaRCa1* (Fig. 6), suggesting a cluster of Colletotrichum resistance loci on LG 6B. Further analyses are required to detect possible associations of alleles from these two loci that may influence simultaneous selection for both resistances.

The *FaRCa1* locus appears distinct from the *Rca2* locus described by Denoyes-Rothan et al. (2005). This conclusion is supported by both physical and genetic mapping approaches. First, the CAC_240_2 and CAC_417_3 reverse markers linked to *Rca2* (Lerceteau-Kohler et al. 2005) were located to chromosome 7 on the diploid woodland strawberry reference genome. Second, the CAC_240_2 marker from the same study was genetically mapped to LG 7B in ‘Winter Dawn’ × ‘Treasure.’ A third line of evidence distinguishing resistance mediated by *Rca2* and *FaRCa1* relates to recent advancements in the taxonomy of *C. acutatum* as well as previous research on pathogenicity groups. Sreenivasaprasad and Talhinhas (2005) described eight different molecular groups, A1–A8, within the *C. acutatum* species complex, which are pathogenic to strawberry and other plants worldwide. Groups A2 and A4 were dominant in Belgium; however, group A2 appeared to be more aggressive than group A4 to both strawberry fruit and crown of the cultivar Elsanta (Van Hemelrijck et al. 2010). The presence of subgroups within the *C. acutatum* species complex was accepted and recognized using multi-locus molecular phylogenetic analysis of strains from numerous hosts with wide geographic distributions (Damm et al. 2012). Based on this analysis, previously described intraspecific groups were assigned to different species in the complex: A2 (*C. nymphaeae* and *C. simmondsii*), A3 (*C. fioriniae*), A4 (*C. godetiae*), A5 (*C. acutatum*) and A7 (*C. salicis*) (Damm et al. 2012).

Preliminary phylogenetic sequencing analysis based on multi-locus sequence indicates that the vast majority of isolates from the *C. acutatum* complex in the USA belong to the species *C. nymphaeae*, followed by a small portion of isolates that belong to species *C. fioriniae* (Wang and Peres, in preparation). These *C. nymphaeae* isolates phylogenetically grouped with Goff, an isolate collected in the USA and assigned to PG-1 (Denoyes-Rothan et al. 2003). On the other hand, isolates 688b and 1267b collected in France and used to identify *Rca2* are assigned to PG-2 (Denoyes-Rothan et al. 2005). The 1267b isolate shares highest multi-locus sequence similarity with isolates of *C. simmondsii* (Peres and Wang, in preparation). Therefore, our hypothesis is that *FaRCa1*-mediated resistance is effective against PG-1 isolates which correspond to *C. nymphaeae* and that *Rca2*-mediated resistance is effective against PG-2 isolates which correspond to *C. simmondsii*. Further work is needed to adequately test this potential association between pathogenicity groups and newly defined species in the *C. acutatum* complex. Without surveying additional isolates and broader strawberry germplasm, we are not yet able to form any hypotheses about selection for this locus during or subsequent to domestication.

Currently, there are a small number of studies characterizing genes related to anthracnose resistance in strawberry. Casado-Diaz et al. (2006) reported spatial and temporal gene expression profiles after *C. acutatum* infection in different cultivars of strawberry. Pathogenesis-related (PR) genes encoding thaumatin-like proteins and hypersensitive-induced response protein had stronger or more rapid expression patterns in less susceptible cultivars. Also, genes encoding a *β*-1-3 glucanase, a class-1 chitinase, a peroxidase and two leucine-rich repeat (LRR) receptor-like proteins were down-regulated in infected fruit. In another study, Guidarelli et al. (2011) performed micro-array analysis in unripe and ripe strawberry fruit 24 h after their exposure to *C. acutatum*. Many genes encoding PR proteins were found to be up-regulated in both unripe and ripe fruit after infection. Also, genes coding for proteins involved in plant detoxification pathways and in the synthesis of stress-related flavonol and alkaloid compounds were up-regulated in both green and red fruits after inoculation. Future studies will locate candidate genes for *FaRCa1* within the delineated region.

In summary, a partially dominant allele at a major subgenome-specific locus, which we name *FaRCa1*, confers resistance to AFR in UF breeding germplasm when challenged with *C. acutatum* species complex isolates from Florida. Together with *FaRCg1*, this locus appears to be part of a group of Colletotrichum resistance loci on LG 6B. This resistance appears to be effective against PG-1 isolates, which we hypothesize are predominately *C. nymphaeae*, as opposed to *Rca2* which is effective against PG-2 isolates. Strong and consistent phenotypic effects of *FaRCa1* were detected across trials and validated in the parent pool of the UF strawberry breeding program and widely grown cultivars. These results should form the basis for breeding tools that will lead to more efficient selection for AFR resistance in the UF strawberry breeding program and other breeding programs utilizing resistant UF germplasm.

### Author contribution statement

NS performed phenotyping and analysis for the 2016–2017 and 2017–2018 seasons and prepared the initial manuscript; SV prepared the linkage map, provided guidance for QTL analysis and helped with analysis in both seasons; NP provided project and inoculation guidance and assistance; VW initiated the research and provided overall project and analysis guidance. All authors have reviewed and edited the manuscript.

## Electronic supplementary material

Below is the link to the electronic supplementary material.

Supplementary material 1 (DOCX 81 kb)

## References

[CR1] Allard A, Bink MC, Martinez S, Kelner JJ, Legave JM (2016). Detecting QTLs and putative candidate genes involved in budbreak and flowering time in an apple multiparental population. J Exp Bot.

[CR2] Anciro A, Mangandi J, Verma S, Peres N, Whitaker VM, Lee S (2018). *FaRCg1*: a quantitative trait locus conferring resistance to Colletotrichum crown rot caused by *Colletotrichum gloeosporioides* in octoploid strawberry. Theor Appl Genet.

[CR3] Antanaviciute L, Surbanovski N, Harrison N, McLeary KJ, Simpson DW (2015). Mapping QTL associated with *Verticillium dahliae* resistance in the cultivated strawberry (*Fragaria* × *ananassa*). Hortic Res.

[CR4] Bassil NV, Davis TM, Zhang H, Ficklin S, Mittmann M (2015). Development and preliminary evaluation of a 90K Axiom^(R)^ SNP array for the allo-octoploid cultivated strawberry *Fragaria* × *ananassa*. BMC Genom.

[CR5] Bassil NV, Hummer KE, Finn CE (2017). Lessons learned from DNA-based tool development and use in a genebank. Acta Hortic.

[CR6] Bink MC, Uimari P, Sillanpaa J, Janss G, Jansen C (2002). Multiple QTL mapping in related plant populations via a pedigree-analysis approach. Theor Appl Genet.

[CR7] Bink MC, Boer MP, ter Braak CJF, Jansen J, Voorrips RE, van de Weg WE (2008). Bayesian analysis of complex traits in pedigreed plant populations. Euphytica.

[CR8] Bink MC, Totir LR, ter Braak CJ, Winkler CR, Boer MP, Smith OS (2012). QTL linkage analysis of connected populations using ancestral marker and pedigree information. Theor Appl Genet.

[CR9] Bink MC, Jansen J, Madduri M, Voorrips RE, Durel CE (2014). Bayesian QTL analyses using pedigreed families of an outcrossing species, with application to fruit firmness in apple. Theor Appl Genet.

[CR10] Cai L, Voorrips RE, van de Weg E, Peace C, Iezzoni A (2017). Genetic structure of a QTL hotspot on chromosome 2 in sweet cherry indicates positive selection for favorable haplotypes. Mol Breed.

[CR11] Casado-Diaz A, Encinas-Villarejo S, de los Santos B, Schiliro E, Yubero-Serrano EM (2006). Analysis of strawberry genes differentially expressed in response to *Colletotrichum* infection. Physiol Plant.

[CR12] Chandler CK, Mertely JC, Peres N (2006). Resistance of selected strawberry cultivars to anthracnose fruit rot and botrytis fruit rot. Acta Hortic.

[CR13] Clark MD, Schmitz CA, Rosyara UR, Luby JJ, Bradeen JM (2014). A consensus ‘Honeycrisp’ apple (*Malus* × *domestica*) genetic linkage map from three full-sib progeny populations. Tree Genet Genom.

[CR14] Damm U, Cannon PF, Woudenberg JHC, Crous PW (2012). The *Colletotrichum acutatum* species complex. Stud Mycol.

[CR15] Denoyes-Rothan B, Guérin G, Délye C, Smith C, Minz D, Maymon M, Freeman S (2003). Genetic diversity and pathogenic variability among isolates of *Colletotrichum* species from strawberry. Phytopathology.

[CR16] Denoyes-Rothan B, Lerceteau-Köhler E, Guérin G, Bosseur S, Bariac J, Martin E, Roudeillac P (2004). QTL analysis for resistances to *Colletotrichum acutatum* and *Phytophthora cactorum* in octoploid strawberry (*Fragaria* × *ananassa*). Acta Hortic.

[CR17] Denoyes-Rothan B, Guerin G, Lerceteau-Kohler E, Risser G (2005). Inheritance of resistance to *Colletotrichum acutatum* in *Fragaria* × *ananassa*. Phytopathology.

[CR18] Di Guardo M, Bink M, Guerra W, Letschka T, Lozano L (2017). Deciphering the genetic control of fruit texture in apple by multiple family-based analysis and genome-wide association. J Exp Bot.

[CR19] Durand JB, Allard A, Guitton B, van de Weg E, Bink M, Costes E (2017). Predicting flowering behavior and exploring its genetic determinism in an apple multi-family population based on statistical indices and simplified phenotyping. Front Plant Sci.

[CR20] Edger PP, VanBuren R, Colle M, Poorten TJ, Wai CM (2018). Single-molecule sequencing and optical mapping yields an improved genome of woodland strawberry (*Fragaria vesca*) with chromosome-scale contiguity. GigaScience.

[CR21] Forcelini BB, Seijo TE, Amiri A, Peres NA (2016). Resistance in strawberry isolates of *Colletotrichum acutatum* from Florida to quinone-outside inhibitor fungicides. Plant Dis.

[CR22] Forcelini BB, Gonçalves FP, Peres NA (2017). Effect of inoculum concentration and interrupted wetness duration on the development of anthracnose fruit rot of strawberry. Plant Dis.

[CR23] Fresnedo-Ramirez J, Bink MC, van de Weg E, Famula TR, Crisosto CH (2015). QTL mapping of pomological traits in peach and related species breeding germplasm. Mol Breed.

[CR24] Fresnedo-Ramirez J, Frett TJ, Sandefur PJ, Salgado-Rojas A, Clark JR (2016). QTL mapping and breeding value estimation through pedigree-based analysis of fruit size and weight in four diverse peach breeding programs. Tree Genet Genom.

[CR25] Guan YZ, Peace C, Rudell D, Verma S, Evans K (2015). QTLs detected for individual sugars and soluble solids content in apple. Mol Breed.

[CR26] Guidarelli M, Carbone F, Mourgues F, Perrotta G, Rosati C, Bertolini P, Baraldi E (2011). *Colletotrichum acutatum* interactions with unripe and ripe strawberry fruits and differential responses at histological and transcriptional levels. Plant Pathol.

[CR27] Haymes KM, van de Weg WE, Arens P, Maas JL, Vosman B, Den Nijs APM (2000). Development of SCAR markers linked to a *Phytophthora fragariae* resistance gene and their assessment in European and North American strawberry genotypes. J Am Soc Hortic Sci.

[CR28] Hernández-Mora JR, Micheletti D, Bink MC, van de Weg E, Cantín C (2017). Integrated QTL detection for key breeding traits in multiple peach progenies. BMC Genom.

[CR29] Hernández-Mora JR, Micheletti D, Bink MC, van de Weg WE, Bassi D (2017). Discovering peach QTLs with multiple progeny analysis. Acta Hortic.

[CR30] Howard CM, Maas JL, Chandler CL, Albregts EA (1992). Anthracnose of strawberry caused by the *Colletotrichum* complex in Florida. Phytopathology.

[CR31] Howard N, van de Weg E, Tillman J, Tong C, Silverstein K, Luby J (2017). Two QTL characterized for soft scald and soggy breakdown in apple (*Malus *× *domestica*) through pedigree-based analysis of a large population of interconnected families. Tree Genet Genom.

[CR32] Iezzoni A, Peace C, Main D, Bassil N, Coe M, Finn C, Gasic K, Luby J, Hokanson S, McFerson J, Norelli J, Olmstead M, Whitaker V, Yue C (2017). RosBREED2: progress and future plans to enable DNA-informed breeding in the Rosaceae. Acta Hortic.

[CR33] Keb-Llanes M, González G, Chi-Manzanero B, Infante D (2002). A rapid and simple method for small-scale DNA extraction in *Agavaceae* and other tropical plants. Plant Mol Biol Rep.

[CR34] Lerceteau-Kohler E, Guerin G, Denoyes-Rothan B (2005). Identification of SCAR markers linked to *Rca2* anthracnose resistance gene and their assessment in strawberry germplasm. Theor Appl Genet.

[CR35] Lerceteau-Kohler E, Moing A, Guerin G, Renaud C, Petit A, Rothan C, Denoyes B (2012). Genetic dissection of fruit quality traits in the octoploid cultivated strawberry highlights the role of homoeo-QTL in their control. Theor Appl Genet.

[CR36] Lipka A, Tian F, Wang Q, Peiffer J, Li M, Bradbury PJ, Gore MA, Buckler ES, Zhang Z (2012). GAPIT: genome association and prediction integrated tool. Bioinformatics.

[CR37] MacKenzie SJ, Peres NA, Barquero MP, Arauz LF, Timmer LW (2009). Host range and genetic relatedness of *Colletotrichum acutatum* isolates from fruit crops and leatherleaf fern in Florida. Phytopathology.

[CR38] Mangandi J, Verma S, Osorio L, Peres NA, van de Weg E, Whitaker VM (2017). Pedigree-based analysis in a multiparental population of octoploid strawberry reveals QTL alleles conferring resistance to *Phytophthora cactorum*. G3 (Bethesda, Md).

[CR39] Mertely JC, Peres NA (2012). Anthracnose fruit rot of strawberry. Univ Fla IFAS EDIS.

[CR40] Mertely JC, Forcelini BB, Peres NA (2017). Anthracnose fruit rot of strawberry. Univ Fla IFAS EDIS.

[CR41] Moose SP, Mumm RH (2008). Molecular plant breeding as the foundation for 21st century crop improvement. Plant Physiol.

[CR42] Peres NA, Timmer LW, Adaskaveg JE, Correll JC (2005). Lifestyles of *Colletotrichum acutatum*. Plant Dis.

[CR100] R Core Team (2013) R: a language and environment for statistical computing. R Foundation for Statistical Computing, Vienna. http://www.R-project.org

[CR43] Roach JA, Verma S, Peres NA, Jamieson AR, van de Weg WE (2016). *FaRXf1*: a locus conferring resistance to angular leaf spot caused by *Xanthomonas fragariae* in octoploid strawberry. Theor Appl Genet.

[CR44] Rosyara UR, Bink MC, van de Weg E, Zhang GR, Wang DC (2013). Fruit size QTL identification and the prediction of parental QTL genotypes and breeding values in multiple pedigreed populations of sweet cherry. Mol Breed.

[CR45] Seijo TE, Chandler C, Mertely J, Moyer C, Peres NA (2008). Resistance of strawberry cultivars and advanced selections to anthracnose and botrytis fruit rots. Proc Fla State Hortic Soc.

[CR46] Seijo TE, Mertely J, Whitaker VM, Peres NA (2012). Evaluation of strawberry cultivars and advanced selections for resistance to anthracnose and botrytis fruit rots, 2010–11. Plant Dis Manag Rep.

[CR47] Seijo TE, Mertely J, Whitaker VM, Peres NA (2012). Evaluation of strawberry cultivars and advanced selections for resistance to anthracnose and botrytis fruit rots and angular leaf spot, 2011–12. Plant Dis Manag Rep.

[CR48] Shulaev V, Sargent DJ, Crowhurst RN, Mockler TC, Folkerts O (2011). The genome of woodland strawberry (*Fragaria vesca*). Nat Genet.

[CR49] Simpson DW, Winterbottom CQ, Bell JA, Maltoni ML (1994). Resistance to a single UK isolate of *Colletotrichum acutatum* in strawberry germplasm from Northern Europe. Euphytica.

[CR50] Smith BJ (1990). Morphological, cultural, and pathogenic variation among *Colletotrichum* species isolated from strawberry. Plant Dis.

[CR51] Sreenivasaprasad S, Talhinhas P (2005). Genotypic and phenotypic diversity in *Colletotrichum acutatum*, a cosmopolitan pathogen causing anthracnose on a wide range of hosts. Mol Plant Pathol.

[CR52] Tennessen JA, Govindarajulu R, Ashman TL, Liston A (2014). Evolutionary origins and dynamics of octoploid strawberry subgenomes revealed by dense targeted capture linkage maps. Genome Biol Evol.

[CR53] van de Weg E, Voorrips RE, Finkers HJ, Kodde LP, Jansen J, Bink MC (2004). Pedigree genotyping: a new pedigree-based approach of QTL identification and allele mining. Acta Hortic.

[CR54] van de Weg E, Bink MC, Voorrips RE, Kruisselbrink JW, Jansen H (2015). Pedigree based analyses: a powerful approach for QTL discovery in pedigreed breeding germplasm and support on breeding decisions. Contemp Hortic.

[CR55] van de Weg E, Di Guardo M, Jänsch M, Socquet-Juglard D, Costa F (2017). Epistatic fire blight resistance QTL alleles in the apple cultivar ‘Enterprise’ and selection X-6398 discovered and characterized through pedigree-informed analysis. Mol Breed.

[CR56] van Dijk T, Pagliarani G, Pikunova A, Noordijk Y, Yilmaz-Temel H (2014). Genomic rearrangements and signatures of breeding in the allo-octoploid strawberry as revealed through an allele dose based SSR linkage map. BMC Plant Biol.

[CR57] Van Hemelrijck W, Debode J, Heungens K, Maes M, Creemers P (2010). Phenotypic and genetic characterization of *Colletotrichum* isolates from Belgian strawberry field. Plant Pathol.

[CR58] Van Ooijen JW (2006). JoinMap^®^ 4.0: software for the calculation of genetic linkage maps in experimental population.

[CR59] Van Ooijen JW (2011). Multipoint maximum likelihood mapping in a full-sib family of an outbreeding species. Genet Res.

[CR60] Verma S, Bassil NV, van de Weg E, Harrison RJ, Monfort A (2017). Development and evaluation of the Axiom^®^ IStraw35 384HT array for the allo-octoploid cultivated strawberry *Fragaria* × *ananassa*. Acta Hortic.

[CR61] Verma S, Zurn JD, Salinas N, Mathey MM, Denoyes B (2017). Clarifying sub-genomic positions of QTLs for flowering habit and fruit quality in U.S. strawberry (*Fragaria* ×* ananassa*) breeding populations using pedigree-based QTL analysis. Hortic Res.

[CR62] Voorrips RE, Bink MC, van de Weg WE (2012). Pedimap: software for the visualization of genetic and phenotypic data in pedigrees. J Hered.

[CR63] Weebadde CK, Wang D, Finn CE, Lewers KS, Luby JJ (2007). Using a linkage mapping approach to identify QTL for day-neutrality in the octoploid strawberry. Plant Breed.

[CR64] Whitaker VM, Osorio LF, Hasing T (2012). Estimation of genetic parameters for 12 fruit and vegetative traits in the University of Florida strawberry breeding population. J Am Soc Hortic Sci.

[CR65] Whitaker VM, Lee S, Osorio LF, Verma S, Roach JA (2017). Advances in strawberry breeding at the University of Florida. Acta Hortic.

[CR66] Xu Y, Li P, Yang Z, Xu C (2017). Genetic mapping of quantitative trait loci in crops. Crop J.

[CR67] Zorrilla-Fontanesi Y, Rambla JL, Cabeza A, Medina JJ, Sanchez-Sevilla JF (2012). Genetic analysis of strawberry fruit aroma and identification of *O*-*methyltransferase FaOMT* as the locus controlling natural variation in mesifurane content. Plant Physiol.

