# Supplementary material for: FaRCa1: a major subgenome-specific locus conferring resistance to Colletotrichum acutatum in strawberry
Source: Theor Appl Genet. 2018 Dec 18;132(4):1109–20. doi: 10.1007/s00122-018-3263-7 (PMC6449309; doi:10.1007/s00122-018-3263-7)
Supplement: Supplementary file 1 — Supplementary material 1 (DOCX 81 kb) [file 122_2018_3263_MOESM1_ESM.docx]

**SUPPLEMENTARY TABLES**

**Supplementary Table 1** Means and standard errors for disease incidences for 77 advanced selections in the UF strawberry breeding program and 10 cultivars tested in 2017-18

| **Order** | **Genotypes** | **Disease Incidence Mean (%)** | **Standard Error** |
| --- | --- | --- | --- |
| 1 | 15.60-15 | 3.42 | 0.85 |
| 2 | 16.2-163 | 4.95 | 1.66 |
| 3 | 15.46-86 | 5.37 | 1.49 |
| 4 | 16.30-143 | 5.49 | 1.44 |
| 5 | ‘Elyana’ | 5.67 | 1.42 |
| 6 | ‘Sweet Charlie’ | 6.29 | 1.48 |
| 7 | 14.83-36 | 6.33 | 2.90 |
| 8 | 16.36-114 | 6.39 | 2.38 |
| 9 | 16.36-131 | 6.44 | 2.21 |
| 10 | 16.40-83 | 7.65 | 2.81 |
| 11 | 16.30-30 | 7.90 | 1.91 |
| 12 | 16.40-73 | 7.99 | 2.41 |
| 13 | 16.36-108 | 8.38 | 2.81 |
| 14 | ‘Florida127’ | 8.76 | 3.28 |
| 15 | 16.13-125 | 9.03 | 2.11 |
| 16 | 13.42-113 | 9.47 | 0.90 |
| 17 | 16.36-123 | 9.52 | 2.56 |
| 18 | 16.13-103 | 9.64 | 2.91 |
| 19 | 12.93-4 | 10.45 | 2.26 |
| 20 | 16.14-47 | 10.52 | 1.75 |
| 21 | 16.40-146 | 11.04 | 2.51 |
| 22 | 16.2-17 | 11.49 | 3.75 |
| 23 | 15.76-60 | 11.71 | 2.80 |
| 24 | 16.30-35 | 11.93 | 2.40 |
| 25 | 16.14-1 | 12.50 | 2.50 |
| 26 | 16.36-10 | 12.51 | 4.15 |
| 27 | 11.58-72 | 12.62 | 2.54 |
| 28 | 16.30-51 | 12.77 | 3.50 |
| 29 | 16.30-87 | 12.84 | 3.26 |

**Supplementary Table 1** Continued

| **Order** | **Genotypes** | **Disease Incidence Mean (%)** | **Standard Error** |
| --- | --- | --- | --- |
| 30 | 14.55-203 | 12.98 | 2.90 |
| 31 | 16.36-37 | 13.01 | 4.67 |
| 32 | 16.30-55 | 13.36 | 4.30 |
| 33 | ‘Radiance’ | 13.58 | 2.85 |
| 34 | 15.81-23 | 13.75 | 3.71 |
| 35 | 16.36-179 | 14.03 | 2.49 |
| 36 | 16.4-22 | 14.03 | 3.87 |
| 37 | 15.66-163 | 14.60 | 2.43 |
| 38 | 13.46-88 | 14.62 | 3.30 |
| 39 | 16.14-42 | 14.68 | 3.52 |
| 40 | 16.13-114 | 15.10 | 2.92 |
| 41 | 16.53-81 | 15.87 | 3.04 |
| 42 | 16.25-154 | 15.88 | 2.00 |
| 43 | 16.30-148 | 16.14 | 3.51 |
| 44 | 15.65-15 | 16.80 | 2.20 |
| 45 | 16.36-44 | 16.83 | 3.46 |
| 46 | 15.65-21 | 17.57 | 1.80 |
| 47 | 16.14-52 | 17.78 | 3.71 |
| 48 | 15.62-7 | 21.24 | 3.62 |
| 49 | 16.40-134 | 21.76 | 3.73 |
| 50 | 16.30-14 | 22.46 | 4.14 |
| 51 | 16.25-171 | 22.80 | 4.12 |
| 52 | 16.2-40 | 22.87 | 4.50 |
| 53 | 16.40-287 | 23.46 | 3.65 |
| 54 | 16.13-50 | 23.59 | 5.20 |
| 55 | 15.35-141 | 24.40 | 3.30 |
| 56 | 15.15-152 | 24.45 | 2.89 |
| 57 | 16.4-46 | 24.77 | 3.72 |
| 58 | 15.35-75 | 24.89 | 1.94 |
| 59 | 16.2-140 | 24.93 | 4.67 |
| 60 | 15.42-130 | 25.21 | 2.69 |

**Supplementary Table 1** Continued

| **Order** | **Genotypes** | **Disease Incidence Mean (%)** | **Standard Error** |
| --- | --- | --- | --- |
| 61 | 13.19-86 | 25.39 | 6.23 |
| 62 | 14.43-47 | 26.24 | 7.18 |
| 63 | 16.13-75 | 26.72 | 4.82 |
| 64 | 15.42-183 | 26.99 | 2.92 |
| 65 | ‘Festival’ | 27.30 | 3.11 |
| 66 | 16.25-165 | 27.57 | 2.32 |
| 67 | 15.9-62 | 27.97 | 3.06 |
| 68 | 16.40-262 | 31.74 | 5.27 |
| 69 | 16.14-34 | 32.94 | 8.81 |
| 70 | 15.35-102 | 33.98 | 6.01 |
| 71 | 15.42-146 | 34.26 | 5.56 |
| 72 | 14.37-103 | 34.91 | 3.79 |
| 73 | 16.30-144 | 35.65 | 3.50 |
| 74 | 15.42-51 | 36.28 | 6.62 |
| 75 | 14.55-48 | 37.77 | 3.06 |
| 76 | 16.30-8 | 37.80 | 4.63 |
| 77 | 15.35-83 | 38.38 | 3.34 |
| 78 | ‘Fronteras’ | 38.67 | 4.27 |
| 79 | 15.63-126 | 39.18 | 10.33 |
| 80 | 16.14-63 | 41.65 | 3.85 |
| 81 | 13.42-5 | 42.46 | 4.92 |
| 82 | ‘Portola’ | 44.59 | 3.02 |
| 83 | ‘Camarosa’ | 46.53 | 4.65 |
| 84 | 16.2-161 | 46.79 | 5.75 |
| 85 | 16.25-43 | 48.89 | 7.26 |
| 86 | ‘Monterey’ | 50.00 | 5.50 |
| 87 | ‘Treasure’ | 59.37 | 5.31 |

**Supplementary Table 2** Means and standard errors for disease incidences of parents, selections and cultivars common in the two discovery populations in 2016-17 and 2017-18, and in the validation population in 2017-18

| **Genotypes** | **Discovery Population 2016-17** | | **Discovery Population 2017-18** | | **Validation Population 2017-18** | |
| --- | --- | --- | --- | --- | --- | --- |
|  | **Disease Incidence Mean (%)** | **Standard Error** | **Disease Incidence Mean (%)** | **Standard Error** | **Disease Incidence Mean (%)** | **Standard Error** |
| ‘Sweet Charlie’ | 16.1 | 2.4 | 8.2 | 2.0 | 6.3 | 1.48 |
| 11.98-41 | 17.0 | 3.5 | 8.0 | 1.8 | - | - |
| ‘Florida127’ | 23.0 | 2.7 | 9.3 | 2.6 | 8.8 | 3.28 |
| Winterdawn | 24.2 | 2.9 | 9.6 | 1.8 | - | - |
| 13.42-113 | 25.1 | 1.4 | - | - | 9.5 | 0.90 |
| ‘Elyana’ | 26.1 | 3.6 | 7.9 | 4.6 | 5.7 | 1.42 |
| 13.51-134 | 29.9 | 5.4 | 12.1 | 3.6 | - | - |
| ‘Brilliance’ | 30.9 | 4.7 | 10.0 | 2.4 | - | - |
| 11.58-72 | 38.4 | 6.9 | - | - | 12.6 | 2.54 |
| 16.25-43 | 48.7 | 9.2 | - | - | 48.9 | 7.26 |
| 13.55-195 | 53.6 | 2.7 | 25.2 | 4.5 | - | - |
| 13.42-5 | 62.2 | 5.5 | 35.8 | 3.0 | 42.5 | 4.92 |
| 12.90-53 | 66.1 | 6.6 | 39.9 | 4.3 | - | - |
| ‘Camarosa’ | 71.4 | 4.0 | 28.6 | 6.3 | 46.5 | 4.65 |
| ‘Treasure’ | 80.6 | 4.9 | 56.5 | 6.3 | 59.4 | 5.31 |
| ‘Festival’ | - | - | 21.7 | 2.8 | 27.3 | 3.11 |
| 13.19-86 | - | - | 41.6 | 5.7 | 25.4 | 6.23 |

**SUPPLEMENTARY FIGURES**


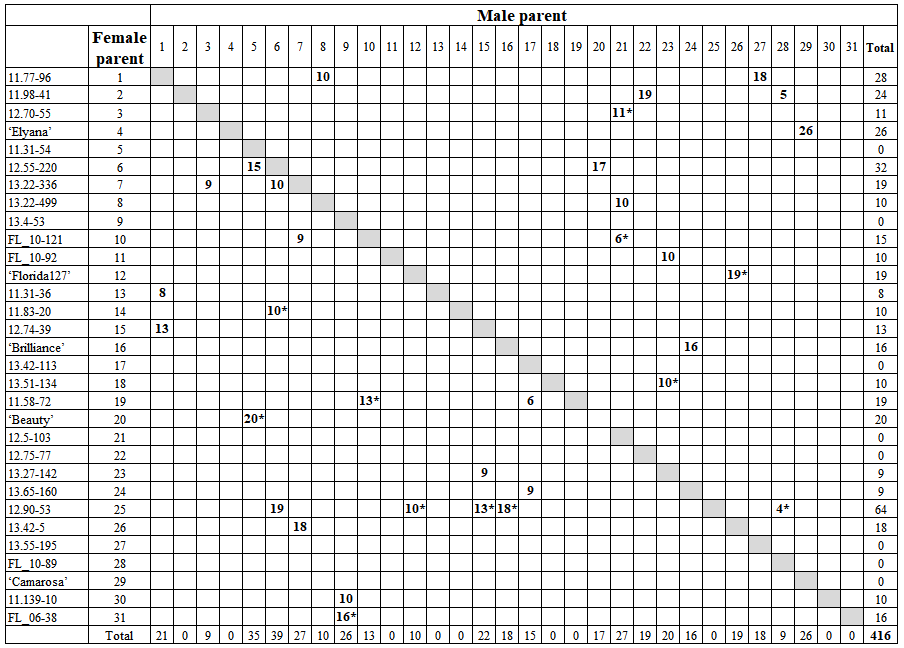


**Supplementary Fig. 1** Schematic representation of a complex connected set of bi-parental crosses from the elite strawberry breeding population at University of Florida (UF) evaluated during the 2016-17 season. The number shown at the intersection of each pair of parents represents the number of seedlings evaluated for the respective cross. The total number of seedlings arising from each parent used as a female and as a male are shown in the far-right column and bottom row, respectively. Families where at least one individual did not have a clear marker segregation based on the parental genotypes are denoted with an asterisk (*)


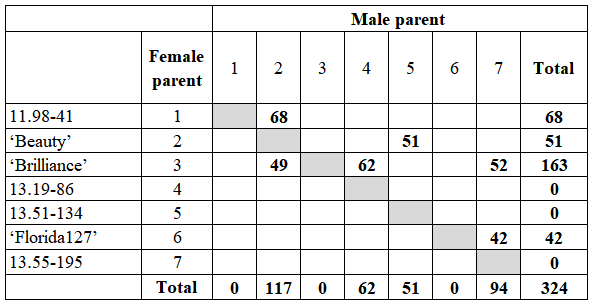


**Supplementary Fig. 2** Schematic representation of six full-sib families representing resistant × susceptible crosses evaluated during the 2017-18 season. The number shown at the intersection of each pair of parents represents the number of seedling individuals evaluated for the respective cross. The total number of seedlings arising from each parent used as a female and as a male are shown in the far-right column and bottom row, respectively
